# N^1^-Methylnicotinamide Improves Hepatic Insulin Sensitivity via Activation of SIRT1 and Inhibition of FOXO1 Acetylation

**DOI:** 10.1155/2020/1080152

**Published:** 2020-03-23

**Authors:** Jingfan Zhang, Yu Chen, Cong Liu, Ling Li, Ping Li

**Affiliations:** Department of Endocrinology, Shengjing Hospital of China Medical University, Shenyang, Liaoning 110004, China

## Abstract

**Objective:**

To explore the effects of N^1^-methylnicotinamide (MNAM) on insulin resistance and glucose metabolism in obese type 2 diabetes mellitus (T2DM) mice and regulatory mechanisms of the NAD-dependent deacetylase sirtuin-1 (SIRT1)/forkhead box protein O1 (FOXO1) pathway.

**Methods:**

Blood glucose and insulin levels were examined in mice. HE and oil red O staining were used to observe the effects of MNAM on liver lipid deposition in ob/ob mice. Real-time PCR and Western blotting were used to detect expression of gluconeogenesis, insulin signaling-related proteins, and SIRT1/FOXO1 pathway-related proteins. L-O2 cells were cultured as a model of insulin resistance, and MNAM and SIRT1 inhibitors were administered *in vivo*. Residual glucose and insulin signaling-related proteins were detected and the mechanisms associated with the SIRT1/FOXO1 signaling pathway in insulin resistance explored.

**Results:**

MNAM can effectively reduce levels of fasting blood glucose and insulin, improve liver morphology, and reduce lipid accumulation in obese type 2 diabetes mellitus mice. MNAM also downregulates the key proteins in the gluconeogenesis pathway in the liver, upregulates *Sirt1* expression, and reduces acetylation of the FOXO1 protein. *In vitro*, MNAM could promote the glucose uptake capacity of L-O2 cells induced by palmitic acid (PA), a saturated fatty acid that induces IR in various scenarios, including hepatocytes, improving insulin resistance. As *Sirt1* expression was inhibited, the reduction of hepatocyte gluconeogenesis and the regulation of the insulin signaling pathway by MNAM were reversed.

**Conclusion:**

MNAM activates SIRT1 and inhibits acetylation of FOXO1, which in turn regulates insulin sensitivity in type 2 diabetic mice, leading to a reduction of hepatic glucose output and improvement of insulin resistance.

## 1. Introduction

Diabetes is one of metabolic diseases characterized by chronic hyperglycemia resulting from a complex etiology, with insulin resistance being one of the most important causes [1]. Patients with type 2 diabetes mellitus (T2DM) account for about 90%-95% of all diabetes cases [2]. The main clinical characteristic of T2DM is insulin resistance accompanied by insulin secretion deficiency [3]. The insulin deficiency and tissue insulin resistance can cause metabolic disorders involving the metabolism of glucose, fat, protein, water, and electrolytes [4]. Long-term high blood glucose levels can cause vascular damage, affecting the function of the heart, eyes, kidneys, and nerves [4, 5]. The clinical treatment of T2DM mainly targets insulin resistance and relative insulin secretion; however, glycemic control in diabetic patients is still hard. Therefore, new therapeutic targets that improve insulin resistance and protect islet *β*-cell function are of great significance for the prevention and treatment of T2DM.

MNAM is a product of methylation of nicotinamide through nicotinamide N-methyltransferase (NNMT) [6]. MNAM has been reported to decrease the concentration of blood glucose and glycosylated hemoglobin and increase C-peptide levels which are slightly elevated in diabetic rats [7]. In a study of NNMT regulation of hepatic nutrition metabolism, Hong et al. [8] reported that intervention with MNAM in high-fat diet mice decreased fasting blood glucose and insulin levels and increased the quantified insulin sensitivity index (QUICKI), suggesting MNAM could prevent changes in fasting blood glucose and insulin induced by a high-fat diet. Therefore, MNAM is beneficial in obesity and insulin resistance; however, the detailed mechanisms are still unknown.

The liver is one of the important target organs for insulin and is the source of 90% of endogenous glucose production [9]. The liver plays an important role in maintaining the production of fasting endogenous glucose, as well as the absorption, utilization, and storage of glucose from food [10]. Expression of the key enzymes in gluconeogenesis is an important marker of hepatic glucose metabolism. During insulin resistance in the liver, the expression of phosphoenolpyruvate carboxykinase (PEPCK) and glucose-6-phosphatase (G-6-Pase) are upregulated significantly. Palmitate (PA) is a saturated fatty acid that induces IR in various scenarios, including hepatocytes [11]. Studies have shown that deacetylase sirtuin-1 (SIRT1) plays an important role in the regulation of liver metabolism [12–14]. SIRT1 agonists can cause the translocation of nuclear transcription factor FOXO1 in liver cells, inhibit gluconeogenesis, and improve insulin sensitivity [15]. This study is aimed at establishing models of obese type 2 diabetes *in vitro* and *in vivo*, observe the effect of MNAM on hepatic insulin sensitivity and glucose metabolism, and explore the regulation of the SIRT1/FOXO1 pathway.

## 2. Materials and Methods

### 2.1. Laboratory Animals and Experimental Design

Male C57BL/6 mice (*n* = 20) and ob/ob mice (*n* = 30), weighing 20 ± 5 g, were purchased from Beijing Huafukang Biotechnology Co. Ltd. Animal experiments were performed in the animal facility of Shengjing Hospital of China Medical University and approved by the Animal Ethics Committee of Shengjing Hospital (approval number: 2016PS340K). C57BL/6 mice were randomly divided into a control group and an MNAM high-dose drug control group (CMNAM), 10 mice each. ob/ob mice were randomly divided into a diabetic model group (DM), MNAM low-dose group (MNAML), and MNAM high-dose group (MNAMH), 10 mice each. Mice in the MNAML and MNAMH groups were treated with MNAM (TCI, Shanghai, China) at a concentration of 0.3% or 1% in dairy food as described in a previous study [8]; mice in the other groups were fed with normal dairy food for 8 weeks.

### 2.2. Assessment of Body Weight and Fasting Blood Glucose (FBG)

After MNAM treatment, mice were weighed every 2 weeks and the FBG was determined every 2 weeks after fasting for 12 hours. Blood was collected from the tip of the tail, and FBG was measured by the CONTOUR®PLUS Blood Glucose Monitoring System 7600P (Bayer, Germany).

### 2.3. Intraperitoneal Glucose Tolerance Test (IPGTT) and Insulin Release Test

After feeding for eight weeks, mice were subjected to an intraperitoneal glucose tolerance test (IPGTT) and insulin release test. Mice were intraperitoneally injected with 1 mg/g glucose (Otsuka, China) [16]. Blood was collected at 0 min, 15 min, 30 min, 60 min, and 120 min, and blood glucose was measured. Blood was separated and the insulin content detected by ELISA (SAB, USA). The blood glucose and insulin content of each group at each time point were plotted, and the area under the curve (AUC) was calculated using the trapezoidal method. The evaluation of insulin resistance consists of three components: firstly, insulin resistance is estimated using the homeostasis model for assessment of insulin resistance (HOMA-IR), which utilizes the following formula: FBG (mmol/L) × fasting insulin level (FINS, mIU/L)/22.5; secondly, QUICKI is calculated from FBG and insulin: 1/(log FBG (mg/dL) and log FINS (mIU/L), which is more correlated with the gold standard positive glucose clamp test used for evaluating insulin resistance. QUICKI is superior to HOMA-IR in calculating fasting insulin resistance [17]. Thirdly, the Matsuda index is calculated from IPGTT glucose and insulin using the following formula:

(1) $$\begin{array}{c} \frac{10000}{\sqrt{\left[\text{FPG}\left(\text{mmol}/\text{L}\right)\times \text{FINS}\left(\mu \text{U}/\text{mL}\right)\right]\times \left[\text{mean GLU OGIRT}\left(\text{mmol}/\text{L}\right)\times \text{mean INS OGIRT}\left(\mu \text{U}/\text{mL}\right)\right]}}. \end{array}$$

The Matsuda index can reflect the overall degree of insulin resistance and is a reliable indicator of reactive insulin resistance tested by the positive glucose clamp technique [18].

### 2.4. Hematoxylin and Eosin Staining

After 8 weeks, the mice were euthanized, and tissues of the mice were fixed in 4% neutral-buffered formaldehyde for 24 h and put in paraffin. 5 *μ*m sections were deparaffinized and stained by hematoxylin and eosin (HE) staining according to the standard HE protocol [19].

### 2.5. Oil Red O Staining

Samples mentioned above in wax were sliced and stained in oil red O dye solution (Solarbio, Beijing, China) for 10 minutes. The slices were immersed in 60% isopropanol for 2 s, then distilled water for 1 s. Slices were restained with hematoxylin for 2 mins and washed with distilled water. A water-soluble sealer was used to fix and seal, and the liver fat droplets and sections were observed under a microscope.

### 2.6. Real-Time PCR Assay

The relative expression levels of mRNA of liver *Pepck* and *G-6-Pase* were measured by real-time PCR. Primers were designed using DNASTAR software according to genetic sequences as reported in GenBank (Table 1). RNA was obtained by the TRIzol method, and first-strand cDNA was synthesized by reverse transcription (Thermo, K1622). Real-time PCR was performed using the qRT-PCR kit (Qiagen, 204057). The reaction conditions were 95°C 30 s, 95°C 5 s, and 0°C 20 s, for 40 cycles; melting curve analysis: 95°C 1 s, 65°C 15 s, and 95°C 5 s. Relative mRNA expression was expressed as 2^-*ΔΔ*CT^.

### 2.7. L-O2 Cell Culture Experiments

L-O2 cells were purchased from the cell bank of the Culture Collection Committee of the Chinese Academy of Sciences, cultured in 10% FBS in RPMI-1640 medium. Cells were divided into the control group, palmitic acid (PA) group, PA+MNAM group (MNAM), and SIRT1 inhibitor group (EX-527). In the control group, L-O2 cells were cultivated in 10% FBS in RPMI-1640 medium. PA (Sigma, P9767) was dissolved in cell culture medium to 0.25 mM; L-O2 cells were cultivated in cell culture medium containing 0.25 mM PA, which is the PA group, an insulin-resistant liver cell model group, according to a previous study [20–22] with slight modification. In the MNAM group, L-O2 cells were cultivated in 10% FBS in RPMI-1640 medium containing 0.25 mM PA and 1 mM MNAM. In the EX-527 group, L-O2 cells were cultured with 10% FBS in RPMI-1640 medium containing 0.25 mM PA, 1 mM MNAM, and 30 *μ*M EX-527 (ab141506, Abcam). All groups of cells were incubated at a constant temperature of 37°C with 5% CO2 and saturated humidity for 24 h.

### 2.8. Glucose Residual Content Detection

Cell culture medium was transferred into a 1.5 mL EP tube and centrifuged at 3500 RPM for 5 min. 2 *μ*L supernatant was diluted with 98 *μ*L deionized water. Glucose content was calculated by reading the absorbance value at 505 nm using a glucose test kit (BC2500, Solarbio, Beijing, China) according to the instructions.

### 2.9. Western Blotting

Samples of liver and L-O2 cells were collected and treated with lysis buffer, protease inhibitor, phosphatase inhibitor, and PMSF, then homogenized. The samples were centrifuged at 12000 rpm for 15 min at 4°C. The protein concentration in supernatants was detected by the BCA method (Wanlei, Shenyang, China). Samples of protein were loaded and separated by SDS-PAGE electrophoresis, transferred to PVDF membranes, and blocked with 5% skimmed milk for 1 h. PVDF membranes were then washed in TBST 3 times. Primary antibodies to PEPCK (1 : 1000, ab70359, Abcam), G-6-Pase (1 : 1000, ab83690, Abcam), p-IRS2 (1 : 1000, ab3690, Abcam), IRS2 (1 : 1000, ab134101, Abcam), p-PI3K (1 : 1000, ab182651, Abcam), PI3K (1 : 1000, ab32089, Abcam), p-AKT (1 : 1000, ab38449, Abcam), AKT (1 : 1000, ab8805, Abcam), p-GSK3*β* (1 : 1000, ab68476, Abcam), GSK3*β* (1 : 1000, ab32391, Abcam), SIRT1 (1 : 1000, ab220807, Abcam), FOXO1 (1 : 1000, ab52857, Abcam), Ac-FOXO1 (1 : 1000, MBS9600633, MyBioSource), and GAPDH (1 : 1000, ab181602, Abcam) were added and membranes incubated overnight at 4° C, then washed in TBST, and incubated with a HRP-labeled secondary antibody (1 : 5000) at room temperature for 1 h. Protein bands on the membranes were revealed by an ECL luminescence kit, imaged by a gel imaging system. ImageJ software was used to calculate the grayscale values. Results were presented as densitometric ratios between the protein of interest and the loading control (GAPDH). Experiments were performed in triplicate and repeated three times.

### 2.10. Statistical Analysis

Statistical analysis was performed using SPSS Statistics V22.0 (IBM, California, USA). Data were expressed as mean ± standard deviation, and ANOVA variance analysis was performed for intergroup comparisons, followed by the Tukey procedure. *P* < 0.05 was considered statistically significant. GraphPad Prism 6.0. was used to plot graphs.

## 3. Results

### 3.1. MNAM Reduces Body Weight and Fasting Blood Glucose Levels in Obese Type 2 Diabetic Mice

**Figure 1:**
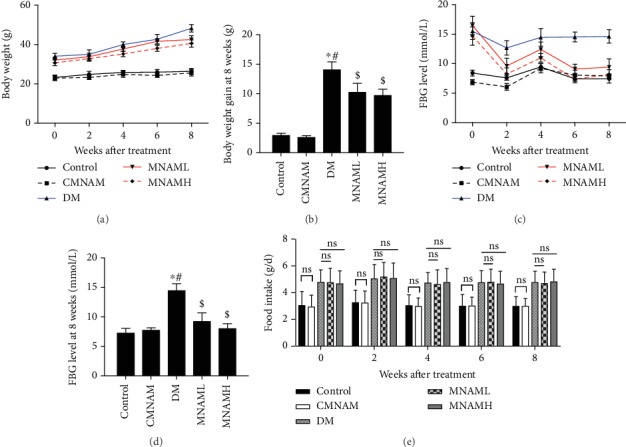
MNAM can reduce body weight and fasting blood glucose levels in obese type 2 diabetic mice (*n* = 10). (a) Weights of mice in each group; (b) weights of mice at 8 weeks in each group; (c) fasting blood glucose of mice in each group; (d) fasting blood glucose of mice at 8 weeks in each group. ^∗^Compared with the control group, *P* < 0.05; ^#^compared with the CMNAM group, *P* < 0.05; ^$^compared with the DM group, *P* < 0.05. (e) Average daily food intake of mice at 8 weeks in each group. ns: *P* > 0.05.

As shown in Figure 1(a), after 8 weeks on normal dairy, the body weight of ob/ob mice in the DM, MNAML, and MNAMH groups increased significantly over time compared with the control group at the beginning of MNAM administration (0 week). As shown in Figure 1(c), fasting blood glucose levels of ob/ob mice in the DM, MNAML, and MNAMH groups were significantly higher than those of mice in the control group (0 week). As the blood glucose of ob/ob mice was more than 13.8 mmol/L [23], a type 2 diabetes mouse model was established successfully. Subsequently, mice were given normal feed or fed with different concentrations of MNAM, and body weight and fasting blood glucose were measured every 2 weeks. As shown in Figure 1, the weights of each group of ob/ob mice (Figure 1(a)) and FBG (Figure 1(c)) were significantly higher than those of C57BL6 mice (*P* < 0.05). The weights and FBG in the DM group increased significantly. Under treatment with MNAM, mice gained weight slowly and FBG was decreased, the difference more significant with the high dose of MNAM treatment. At 8 weeks, comparing with the DM group without treatment of MNAM, we found that the MNAMH group had significantly lower weight gain (Figure 1(b)) and decreased fasting blood glucose (Figure 1(d), *P* < 0.05 vs. DM). Moreover, the MNAM treatment through food was not observed to affect the daily food intake of C57BL/6 and ob/ob mice (Figure 1(e), *P* > 0.05).

### 3.2. MNAM Enhances Insulin Sensitivity in Obese Type 2 Diabetic Mice

**Figure 2:**
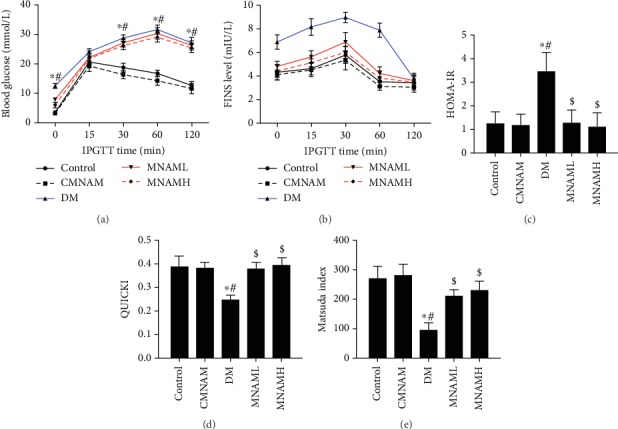
MNAM enhances insulin sensitivity in obese type 2 diabetic mice (*n* = 10). (a) Blood glucose after IPGTT of mice in each group; (b) insulin levels after IPGTT of mice in each group; (c) HOMA-IR data; (d) insulin sensitivity index—QUICKI data; (e) Matsuda index data. ^∗^Compared with the control group, *P* < 0.05; ^#^compared with the CMNAM group, *P* < 0.05; ^$^compared with the DM group, *P* < 0.05.

Using the results of IPGTT and insulin measurement (Figures 2(a) and 2(b)), the insulin resistance-related observation indexes were calculated. The insulin resistance index HOMA-IR of the DM group was significantly higher than that of the control, CMNAM, MNAML, and MNAMH groups (*P* < 0.05). There was no significant difference in HOMA-IR between the control, CMNAM, MNAML, and MNAMH groups (Figure 2(c), *P* > 0.05). The insulin sensitivity index QUICKI of the DM group was significantly lower than that of other groups (*P* < 0.05), and there was no statistical difference between the control, CMNAM, MNAML, and MNAMH groups (Figure 2(d), *P* > 0.05). The Matsuda index was calculated from multiple time points of glucose and insulin after IPGTT and is a reliable indicator of overall insulin resistance. The Matsuda index in the DM group was significantly lower than that in the other groups (*P* < 0.05). After MNAM exposure, the Matsuda index of mice was slightly lower than that of the control and CMNAM groups, but the difference was not statistically significant (Figure 2(e), *P* > 0.05). The data suggested that MNAM can improve insulin sensitivity in T2DM mice.

### 3.3. MNAM Improves Liver Morphology and Reduces Lipid Accumulation in the Liver of Obese T2DM Mice

**Figure 3:**
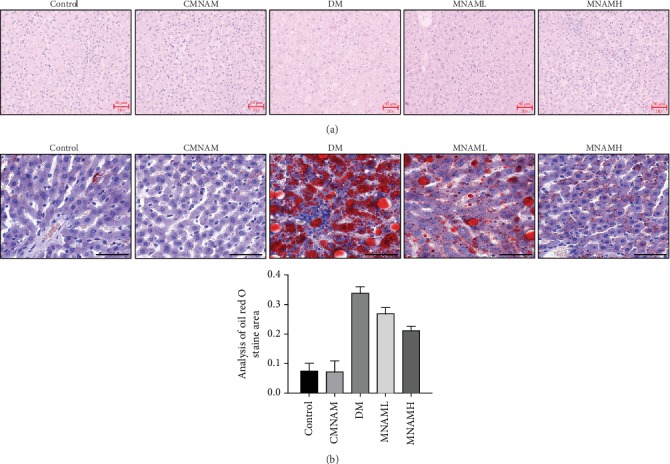
MNAM effectively improves liver morphology and reduces lipid accumulation in the liver (*n* = 10). (a) HE staining (scale bar = 50 *μ*m); (b) oil red staining (scale bar = 50 *μ*m).

HE staining was used to observe histopathological changes in the livers of each group (Figure 3(a)). Liver tissue in the control and CMNAM groups was intact; hepatocytes were evenly distributed and arranged neatly to display the hepatic cords, which were radially distributed. In the DM group, the hepatic cord structure was disordered, the arrangement was irregular, the border of the hepatic lobule was blurred, and most of the hepatocytes were enlarged and showed fatty degeneration. The formation of lipid droplets was further observed after oil red staining (Figure 3(b)), with a large number of orange-red lipid droplets being observed in the DM group. MNAM could significantly improve the liver morphology of T2DM mice, inducing an arrangement of liver cells that was more regular, with reduced fatty degeneration and reduced aggregation of red lipid droplets as shown by oil red staining.

### 3.4. MNAM Reduces Gluconeogenesis in the Liver of Obese T2DM Mice

**Figure 4:**
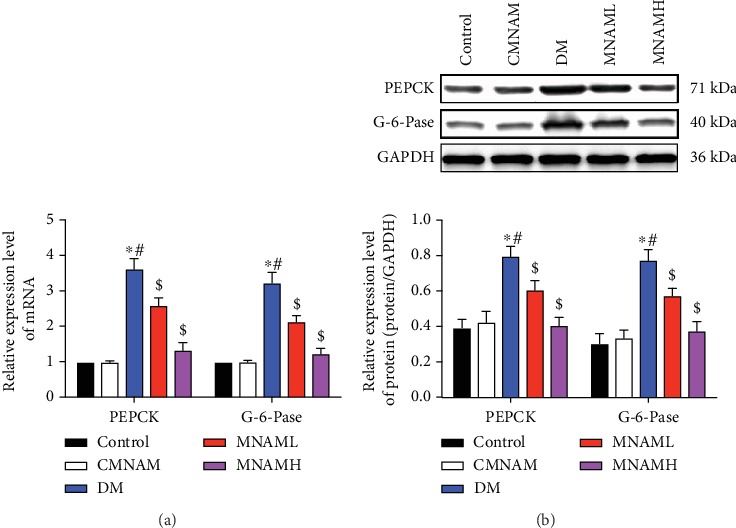
MNAM reduces gluconeogenesis in the liver of obese T2DM mice (*n* = 10). (a) Real-time PCR was used to detect the mRNA expression of *Pepck* and G-6-Pase in the liver tissue of each group; (b) the expression of PEPCK and G-6-Pase in the liver of mice detected by Western blot. ^∗^Compared with the control group, *P* < 0.05; ^#^compared with the CMNAM group, *P* < 0.05; ^$^compared with the DM group, *P* < 0.05.

PEPCK and G-6-Pase are biomarkers of gluconeogenesis in the liver. Therefore, real-time PCR (Figure 4(a)) and Western blotting (Figure 4(b)) were used to detect the mRNA and the protein expression of PEPCK and G-6-Pase in the liver tissues of each group. Compared with the control group, mRNA and protein levels of PEPCK and G-6-Pase in the DM group were significantly increased (*P* < 0.05). After MNAM treatment, PEPCK and G-6-Pase in liver tissue were downregulated in a dose-dependent manner. These results demonstrate that MNAM can reduce the expression of key gluconeogenesis proteins in obese T2DM mice.

### 3.5. MNAM Enhances Protein Expression of the Hepatic Insulin Signaling Pathway

**Figure 5:**
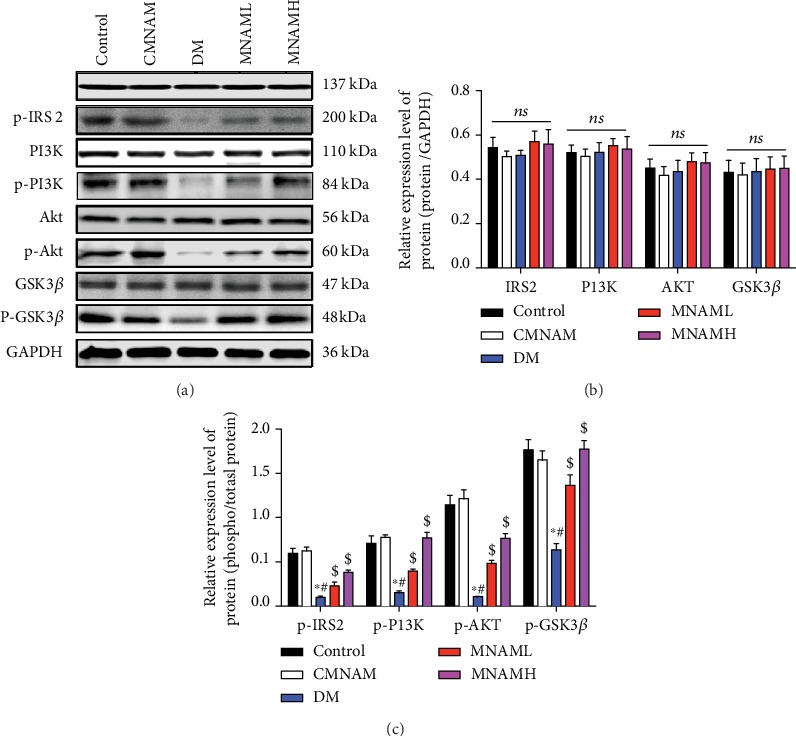
MNAM enhances protein expression of the hepatic insulin signaling pathway. Protein expression and phosphorylation levels of IRS2, PI3K, AKT, and GSK3*β*, of the hepatocyte insulin signaling pathway, measured by Western blot (*n* = 3). (a) Visualization of Western blots; (b) bar graphs of IRS2, PI3K, AKT, and GSK3*β*; (c) bar graphs of p-IRS2/IRS2, p-PI3K/PI3K, p-AKT/AKT, and p-GSK3*β*/GSK3*β*. ^∗^Compared with the control group, *P* < 0.05; ^#^compared with the CMNAM group, *P* < 0.05; ^$^compared with the DM group, *P* < 0.05; ns: not significant, *P* > 0.05.

In order to further investigate the therapeutic effect of MNAM on insulin resistance, we examined protein expression and phosphorylation levels of IRS2, PI3K, AKT, and GSK3*β*, part of the hepatocyte insulin signaling pathway, by Western blot (Figure 5). Levels of p-IRS2/IRS2, p-PI3K/PI3K, p-AKT/AKT, and p-GSK3*β*/GSK3*β* were significantly lower in the DM group (*P* < 0.05). After a low dose and a high dose of MNAM treatment, levels of p-IRS2/IRS2, p-PI3K/PI3K, p-AKT/AKT, and p-GSK3*β*/GSK3*β* were significantly increased (*P* < 0.05 vs. DM). These results suggested that MNAM may improve insulin sensitivity of the liver in T2DM mice, and this may be related to the regulation of the insulin-related signaling pathway.

### 3.6. MNAM Activates *Sirt1* Expression and Inhibits Acetylation of FOXO1 in the Liver of Obese T2DM Mice

**Figure 6:**
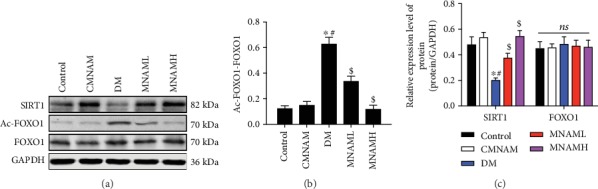
MNAM activates *Sirt1* expression and inhibits acetylation of FOXO1 in the liver of obese T2DM mice (*n* = 3). (a) Western blots; (b) bar graphs of acetylation of FOXO1; (c) bar graphs of SIRT1 and FOXO1. ^∗^Compared with the control group, *P* < 0.05; ^#^compared with the CMNAM group, *P* < 0.05; ^$^compared with the DM group, *P* < 0.05; ns: not significant, *P* > 0.05.

SIRT1 agonists can lead to nuclear translocation of FOXO1 in hepatocytes, inhibiting gluconeogenesis and improving insulin sensitivity. After treatment of T2DM mice with MNAM, the expressions of SIRT1, FoxO1, and acetylation of FOXO1 were detected by Western blot (Figure 6(a)). The ratio of Ac-FOXO1/FOXO1 is shown in Figure 6(b). Relative expression levels of *Sirt1* and *FoxO1* are presented in Figure 6(c). The results showed that the expression of SIRT1 was decreased and the acetylation of FOXO1 increased in the DM group, and the ratio of Ac-FOXO1/FOXO1 was increased in the DM group. Expression of *Sirt1* was significantly upregulated, and acetylation of FOXO1 and the ratio of Ac-FOXO1/FOXO1 were significantly reduced after MNAM administration (*P* < 0.05 vs. DM).

### 3.7. MNAM Regulates Hepatocyte Insulin Sensitivity via Activation of SIRT1

**Figure 7:**
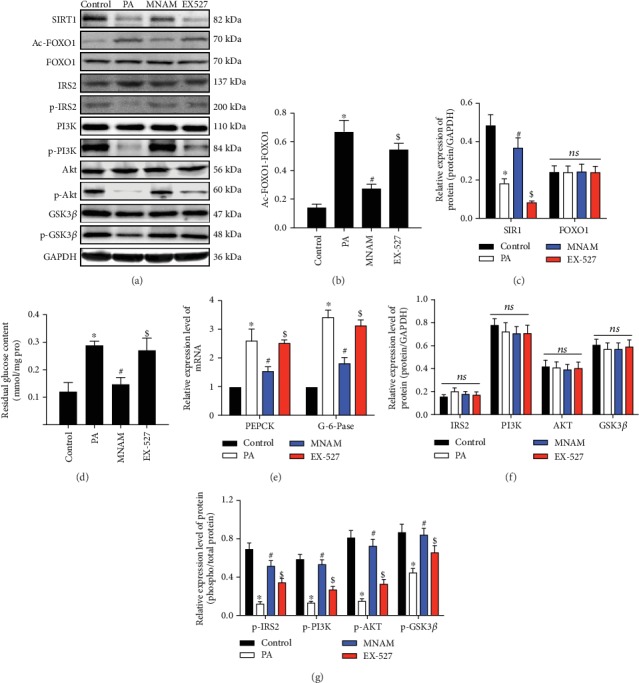
MNAM regulates hepatocyte insulin sensitivity via activation of SIRT1. (a)The result of Western blots (*n* = 3); (b) bar graphs of acetylation of FOXO1; (c) bar graphs of SIRT1 and FOXO1; (d) glucose residual amounts; (e) real-time PCR data used to detect mRNA expression of *Pepck* and *G-6-Pase* in cells from each group; (f) bar graphs of IRS2, PI3K, AKT, and GSK3*β*; (g) bar graphs of p-IRS2/IRS2, p-PI3K/PI3K, p-AKT/AKT, and p-GSK3*β*/GSK3*β*. ^∗^*P* < 0.05, compared with the control group; ^#^*P* < 0.05, compared with the PA group; ^$^*P* < 0.05, compared with the MNAM group; ns: *P* > 0.05, not significant.

PA was used to treat L-O2 cells, as an insulin resistance model [20–22], and the SIRT1/FOXO1 pathway-associated proteins were measured. Data showed that the expression of the SIRT1 protein was decreased and that the level of acetylation of FOXO1 was significantly increased in the PA group, while MNAM treatment upregulated *Sirt1* and downregulated acetylation of FOXO1 (Figures 7(a)–7(c)). Based on *in vivo* and *in vitro* results, we hypothesized that the SIRT1/FOXO1 pathway plays an important role in improving insulin resistance under MNAM treatment. Therefore, we added the SIRT1 inhibitor EX-527 and assessed the amount of glucose remaining in cell culture supernatants of each group. This revealed that compared with the control group, residual glucose in the PA group was significantly increased, glucose content was decreased after the addition of MNAM (Figure 7(d), *P* < 0.05 vs. PA), and EX-527 attenuated the effect of MNAM on insulin-resistant hepatocytes. Residual glucose in the EX-527 group was higher than that in the MNAM group (*P* < 0.05). Real-time PCR was used to detect the relative mRNA expression of *Pepck* and *G-6-Pase*; MNAM downregulated the mRNA of *Pepck* and *G-6-Pase* (*P* < 0.05 vs. PA). mRNA expression of *Pepck* and *G-6-Pase* in the EX-527 group was significantly higher than that in the MNAM group (*P* < 0.05) (Figure 7(e)). Western blotting was used to detect whether inhibition of SIRT1 modulated the effect of MNAM on PA-induced insulin resistance via hepatocyte insulin signaling. Results (Figure 7(a), 7(f), and 7(g)) showed that in the PA group, p-IRS2/IRS2, p-PI3K/PI3K, p-AKT/AKT, and p-GSK3*β*/GSK3*β* were significantly decreased (*P* < 0.05 vs. control). In the MNAM group, the ratios of p-IRS2/IRS2, p-PI3K/PI3K, p-AKT/AKT, and p-GSK3*β*/GSK3*β* were increased; however, in the EX-527 group, the above ratios were decreased. These results showed that MNAM can promote insulin signaling in hepatocytes, while inhibition of SIRT1 could block the effect of MNAM in regulating insulin signaling pathways, in insulin-resistant (IR) hepatocytes.

## 4. Discussion

An important pathogenesis of type 2 diabetes is insulin resistance, which is a key pathological feature of obesity and metabolic syndrome [24]. Increasing insulin sensitivity represents a therapeutic strategy for prevention and treatment of T2DM, obesity, and metabolic syndrome [25, 26]. In this study, we used ob/ob mice to establish an obese type 2 diabetes model and used MNAM as an intervention. We demonstrated that MNAM can regulate liver gluconeogenesis and insulin signaling pathways in obese T2DM mice, reduce liver tissue lipid deposition, and improve insulin resistance in obese T2DM mice, resulting in reduced fasting blood glucose. Our study shows that MNAM can improve insulin sensitivity in obese T2DM mice, and this effect involves activation of the SIRT1/FOXO1 signaling pathway.

Published studies have shown that MNAM can significantly reduce glycosylated hemoglobin and FBG levels [7]. Kuchmerovska et al. confirmed that MNAM can significantly reduce FBG levels in type 1 diabetic rats and slightly lower glycosylated hemoglobin levels, but without effects on nonfasting blood glucose, triglycerides, and total cholesterol [27]. However, Kannt et al. found that MNAM levels were associated with insulin resistance, while plasma MNAM levels were inversely correlated with insulin sensitivity [28]. Plasma MNAM levels were significantly lower in patients who underwent weight loss surgery to improve insulin resistance. In our study, ob/ob mice were used, as the blood glucose reached the level of diabetes, and MNAM intervention was studied. The results showed that the body weights and fasting blood glucose of mice were significantly decreased. To further explore the effect of MNAM on insulin resistance, we demonstrated that MNAM reduces the HOMA-IR index of obese T2DM mice and increases both the QUICKI insulin sensitivity index and the Matsuda index. Therefore, MNAM can improve insulin resistance in obese T2DM mice.

Hepatic insulin resistance is a major cause of abnormal fasting glucose and a key process in the development of T2DM [29]. In the case of complete failure of hepatic insulin receptor signaling, increased gluconeogenesis and the phenomenon of reduced lipogenesis will coexist in the liver, a situation known as “overall insulin resistance” [30]. We showed that MNAM can reduce liver tissue steatosis and lipid droplet infiltration. During insulin resistance in type 2 diabetes, the inhibitory effect of insulin on hepatic gluconeogenesis is reduced, and the expressions of gluconeogenesis proteins PEPCK and G-6-Pase were increased. Therefore, the hepatic glucose output is increased, resulting in an increase in blood glucose. By examining the expression of hepatic gluconeogenesis proteins, we found that MNAM intervention can reduce expression of PEPCK and G-6-Pase in the liver of obese type 2 diabetic mice, indicating that MNAM regulates hepatic gluconeogenesis in T2DM, reduces hepatic glucose output, and improves liver IR.

During the process of liver IR, insulin and its signaling cascade controls cell growth, metabolism, and survival, via stimulation of mitogen-activated protein kinases (MAPKs) and phosphatidylinositol 3-kinase (PI3K), activation of PI3K-associated insulin receptor substrates-1 and 2 (IRS1 and IRS2, respectively), and triggering of the phosphorylation cascade from AKT to FOXO1 [31]. IRS1 mainly affects insulin function in the muscle and fat, while IRS2 plays an important role in the regulation of insulin metabolism in the liver [32]. Simultaneous deletion of IRS1 and IRS2 genes in the liver of mice could inhibit the phosphorylation cascade of AKT, leading to hyperglycemia, hyperinsulinemia, and insulin resistance [33, 34]. Mice with deletions in the PI3K catalytic subunit or AKT2 display insulin resistance and a type 2 diabetic phenotype [3, 35]. Inactivation of liver PI3K, AKT1, or AKT2 could induce hyperglycemia and hyperinsulinemia. In this study, we identified that phosphorylation of insulin signaling pathway-related proteins IRS2 tyrosine, PI3K, AKT, and GSK3*β* was decreased in the ob/ob group, comparable to previous studies [36]. MNAM can increase the phosphorylation of these proteins, increasing activation of insulin signaling pathway-related proteins in the liver of ob/ob T2DM mice and improving liver insulin sensitivity.

The mechanism of MNAM was reported to regulate the metabolic effects of NNMT in the liver. NNMT expression is associated with lower serum cholesterol, triglycerides, and other parameters in both mice and humans. Moreover, MNAM increases SIRT1 protein levels and activity via regulation of ubiquitination of SIRT1, thereby affecting the expression of related proteins of gluconeogenesis; inhibiting the synthesis of fatty acids and cholesterol in hepatocytes; reducing hepatic triglyceride, cholesterol levels, and liver inflammation; and improving liver metabolism [8]. The SIRT1 agonist can improve glucose homeostasis in IR mice by reducing hepatic gluconeogenesis and increasing hepatic insulin sensitivity [37]. In Zucker's fa/fa obese rat model, SIRT1 activating factor (SRT1720) could improve type 2 diabetes by increasing systemic insulin sensitivity and mitochondrial capacity, as well as reducing hepatic glucose production [37]. Inhibition of *Sirt1* expression can upregulate gluconeogenesis-related genes and promote lipogenesis, leading to elevated levels of intracellular lipids and glucose [38]. Our study is the first to demonstrate *in vivo* that MNAM can activate the SIRT1 pathway in ob/ob T2DM mice. However, the hypoglycemic effect of MNAM was weakened by inhibition of the SIRT1 signaling pathway, which promoted hepatic gluconeogenesis, and phosphorylation of insulin signaling pathway-related proteins was not significantly improved. The positive effect of MNAM on insulin sensitivity in diabetes was abrogated, suggesting that the SIRT1/FOXO1 signal pathway could regulate the effects of MNAM in obese T2DM model.

In summary, MNAM plays a role in hepatic insulin resistance in obese T2DM mice. MNAM could reduce body weight and fasting blood glucose levels, increase insulin sensitivity, improve liver morphology, reduce aggregation of lipids in the liver, downregulate the expression of gluconeogenesis-related proteins, and regulate hepatic insulin signaling pathways. This effect is likely achieved via activation of SIRT1 and inhibition of FOXO1 acetylation.

## Figures and Tables

**Table 1:** Primer sequences.

Gene name	Direction	Oligonucleotide sequences
GAPDH	Forward	TGTGTCCGTCGTGGATCTGA
	Reverse	TTGCTGTTGAAGTCGCAGGAG
G6PC	Forward	TGGAGTCTTGTCAGGCATTG
	Reverse	GTAGAATCCAAGCGCGAAAC
PEPCK	Forward	CTGGCACCTCAGTGAAGACA
	Reverse	TCGATGCCTTCCCAGTAAAC

## Data Availability

The data sets used and/or analyzed during the current study are available from the corresponding author on reasonable request.
